# Effects of EEG-based monitoring of depth of anesthesia on postoperative delirium, cognitive dysfunction, and long-term neurocognitive outcomes: a meta-analysis

**DOI:** 10.3389/fmed.2025.1714117

**Published:** 2026-03-12

**Authors:** Xiaochen Huang, Fei Fei

**Affiliations:** 1 Department of Anesthesiology, The Affiliated Jiangning Hospital of Nanjing Medical University, Nanjing Jiangning Hospital, Nanjing, China; 2 Department of Anesthesiology, The Affiliated Huaian Hospital of Xuzhou Medical University, The Second People’s Hospital of Huai’an, Huai’an, China

**Keywords:** depth of anesthesia, meta-analysis, postoperative cognitive dysfunction, postoperative delirium, postoperative complications

## Abstract

**Objective:**

To evaluate the impact of monitoring depth of anesthesia on postoperative delirium, long-term cognitive function, and patient satisfaction through a comprehensive meta-analysis.

**Methods:**

We conducted a systematic review and meta-analysis of studies that assessed the effects of depth of anesthesia monitoring on various postoperative outcomes. Studies were identified through electronic databases, and data were extracted on the duration of anesthesia, early postoperative Mini-Mental State Examination (MMSE) scores, incidence of postoperative delirium, long-term neurocognitive disorders, and patient satisfaction. Pooled standardized mean differences (SMD) and relative risks (RR) were calculated using random-effects models. Heterogeneity was assessed using *I*^2^ statistics, and publication bias was evaluated using funnel plots and Egger’s test.

**Results:**

A total of 17 studies were included, encompassing 5,684 patients. Monitoring depth of anesthesia did not significantly affect the duration of anesthesia or early postoperative MMSE scores. Similarly, no significant difference was observed in the incidence of postoperative delirium before discharge. However, a significant reduction in the incidence of postoperative neurocognitive disorder was noted in long-term follow-up. No significant differences were found in patient satisfaction. High heterogeneity was observed in some analyses, indicating variability across studies.

**Conclusion:**

Monitoring depth of anesthesia appears to have a protective effect against long-term neurocognitive disorders but does not significantly impact short-term cognitive function, delirium incidence, or patient satisfaction. These findings suggest that depth of anesthesia monitoring may be particularly beneficial in high-risk patient populations.

## Introduction

Postoperative delirium (POD) is an acute neuropsychiatric syndrome characterized by fluctuating attention, disorganized thinking, and altered consciousness, affecting 15–50% of elderly surgical patients. Postoperative cognitive dysfunction (POCD), a longer-term decline in memory, executive function, or processing speed, persists in 10–30% of patients beyond 3 months. POD is characterized by acute confusion and altered consciousness, while POCD involves prolonged deficits in memory and executive function (1, 2). Both conditions prolong hospital stays, increase healthcare costs, and elevate mortality risk (3).

The administration of anesthesia is a cornerstone of modern surgical practice, ensuring patient comfort and facilitating optimal operative conditions. However, the balance between adequate sedation and minimizing adverse neurological outcomes remains a critical challenge (4). Postoperative delirium (POD) and long-term cognitive dysfunction, such as postoperative cognitive decline (POCD), are significant complications that impact recovery and quality of life (5). POD is defined as an acute confusional state occurring within 7 postoperative days, diagnosed using validated tools (CAM, CAM-ICU, 3D-CAM, or 4AT), characterized by acute confusion and attention deficits, affects up to 50% of elderly surgical patients, prolonging hospital stays and increasing healthcare costs (6). Beyond the immediate postoperative period, emerging evidence suggests a link between anesthesia exposure and persistent cognitive impairment, raising concerns about its role in accelerating neurodegenerative processes (1).

Depth of anesthesia (DoA) monitoring is defined as the intraoperative use of processed EEG devices (Bispectral Index (BIS), Entropy, Narcotrend, or Patient State Index) where anesthetic administration is actively titrated to maintain indices within manufacturer-recommended or study-specific target ranges (7). Excessive anesthetic depth has been implicated in neuroinflammatory pathways and neuronal apoptosis, potentially exacerbating cognitive deficits (8, 9). By optimizing sedation levels, DoA monitoring may mitigate these risks, offering a promising strategy to enhance postoperative neurological outcomes. Despite this rationale, clinical studies report conflicting results. Some trials demonstrate reduced POD incidence and improved cognitive trajectories with DoA monitoring, while others find no significant benefit, underscoring unresolved controversies in perioperative care. The heterogeneity in existing literature may stem from methodological variations, including differences in monitoring protocols, patient populations, and outcome assessments. For instance, studies often diverge in defining “optimal” anesthesia depth, with thresholds for intervention varying across institutions (10). Additionally, while short-term outcomes like POD are frequently examined, long-term cognitive follow-up remains sparse, leaving gaps in understanding the sustained impact of DoA monitoring. This inconsistency highlights the need for a comprehensive synthesis of evidence to clarify its clinical utility. Despite advances in anesthesia monitoring, conflicting evidence exists regarding the benefits of EEG-guided protocols. While some studies report reduced POD and POCD with light anesthesia, others, including the large ENGAGES trial, found no significant differences. This meta-analysis addresses these discrepancies by evaluating the relationship between anesthesia depth and cognitive outcomes (11, 12).

This meta-analysis addresses these gaps by evaluating the effects of DoA monitoring on two critical endpoints: the incidence of POD and long-term cognitive prognosis. By aggregating data from randomized controlled trials (RCTs) and observational studies, we aim to resolve discrepancies in the literature and explore sources of heterogeneity, such as patient age, surgical type, and anesthesia regimens. Furthermore, we discuss potential mechanisms linking DoA monitoring to cognitive outcomes, including reduced exposure to neurotoxic anesthetic agents and stabilization of intraoperative cerebral perfusion. This meta-analysis evaluates whether active DoA monitoring-guided anesthesia reduces POD incidence, improves long-term cognitive outcomes, and decreases time to emergence compared to standard clinical monitoring in adult patients undergoing non-cardiac, non-intracranial surgery. We also assess the impact of clinical heterogeneity on outcome variability.

## Methods

This meta-analysis was conducted in accordance with the Preferred Reporting Items for Systematic Reviews and Meta-Analyses (PRISMA) guidelines (13). The protocol outlined eligibility criteria, search strategy, and analytical methods to ensure transparency and reproducibility.

### Search strategy and data sources

A comprehensive literature search was performed across four electronic databases: PubMed, Embase, Cochrane Central Register of Controlled Trials (CENTRAL), and Web of Science, from their inception to April 2024. The search strategy combined Medical Subject Headings (MeSH) terms and free-text keywords related to depth of anesthesia (DoA) monitoring, postoperative delirium (POD), and cognitive outcomes. Key terms included: Intervention: “BIS monitoring,” “entropy monitoring,” “depth of anesthesia monitoring,” “EEG-guided anesthesia.” Outcomes: “postoperative delirium,” “POD,” “cognitive dysfunction,” “neurocognitive decline,” “long-term cognition,” “postoperative cognitive decline (POCD).” Population: “surgical patients,” “general anesthesia.” Boolean operators (AND/OR) and database-specific filters were applied to refine results. To minimize publication bias, gray literature sources, including ClinicalTrials.gov, conference proceedings (e.g., Euroanaesthesia), and dissertations, were manually reviewed. Reference lists of included studies and relevant systematic reviews were also screened.

### Study selection and eligibility criteria

Two independent reviewers screened titles, abstracts, and full texts using the web-based tool Rayyan. Discrepancies were resolved through consensus or consultation with a third reviewer.

#### Inclusion criteria

*Population*: patients undergoing elective or emergency surgery under general anesthesia.

*Intervention*: Intraoperative use of DoA monitoring devices [e.g., Bispectral Index (BIS), entropy, Narcotrend] to titrate anesthetic agents.

*Comparator*: Standard anesthesia care without DoA monitoring.

*Outcomes*: Primary outcome: incidence of POD within 7 days post-surgery, diagnosed using validated tools [e.g., Confusion Assessment Method (CAM), DSM-5]. Secondary outcome: long-term cognitive function (≥3 months postoperatively) assessed via standardized neuropsychological tests [e.g., Mini-Mental State Examination (MMSE)].

*Study design*: randomized controlled trials (RCTs) and prospective observational cohort studies.

#### Exclusion criteria

Non-English studies, animal research, case reports, editorials, or studies with insufficient outcome data; trials focusing solely on regional anesthesia or sedation outside the operating room.

### Data extraction and management

A piloted data extraction form was used to collect:

Study characteristics: Author, publication year, country, design, sample size, funding source. Participant demographics: Age, sex, comorbidities (e.g., diabetes, baseline cognitive impairment), surgical type (e.g., cardiac, orthopedic). Intervention details: DoA device type, target index range (e.g., BIS 40–60), anesthesia protocol (volatile vs. intravenous agents, opioid use). Outcome measures: diagnostic criteria for POD, cognitive assessment tools, follow-up duration, attrition rates. Missing data were requested from corresponding authors via email. Two reviewers cross-verified extracted data, with a third reviewer resolving discrepancies.

### Risk of Bias and quality assessment

Risk of bias of included studies quality was appraised using validated tools: the Cochrane Risk of Bias Tool (RoB 2.0) evaluated bias across five domains: randomization process; deviations from intended interventions; missing outcome data; outcome measurement; selective reporting. Studies were classified as low risk, some concerns, or high risk. Assessing the quality of these articles using the Jadad Scale (also known as the Oxford Quality Scoring System) requires evaluating randomized controlled trials (RCTs) across three domains: randomization, blinding, and accounting for withdrawals/dropouts. The maximum score is 5 points, with higher scores indicating better methodological quality. Two reviewers independently performed assessments, achieving >90% inter-rater agreement (kappa statistic = 0.85).

### Statistical analysis

Data were synthesized using Stata software. Dichotomous outcomes (e.g., POD incidence) were pooled as risk ratios (RR) with 95% confidence intervals (CI). Continuous outcomes (e.g., cognitive scores) were analyzed as standardized mean differences (SMD) to account for varying assessment scales. A random-effects model (DerSimonian-Laird method) was employed to address clinical and methodological heterogeneity. Heterogeneity was quantified using I^2^ statistics (I^2^ > 50% indicating substantial heterogeneity) and tau^2^. Subgroup analyses explored heterogeneity sources. Publication bias was evaluated using funnel plots, Egger’s regression test (*p* < 0.10 indicating asymmetry).

### Ethical considerations and data availability

As this study synthesized anonymized, publicly available data, institutional review board approval was not required.

## Results

### Study selection and study characteristics

**Figure 1:**
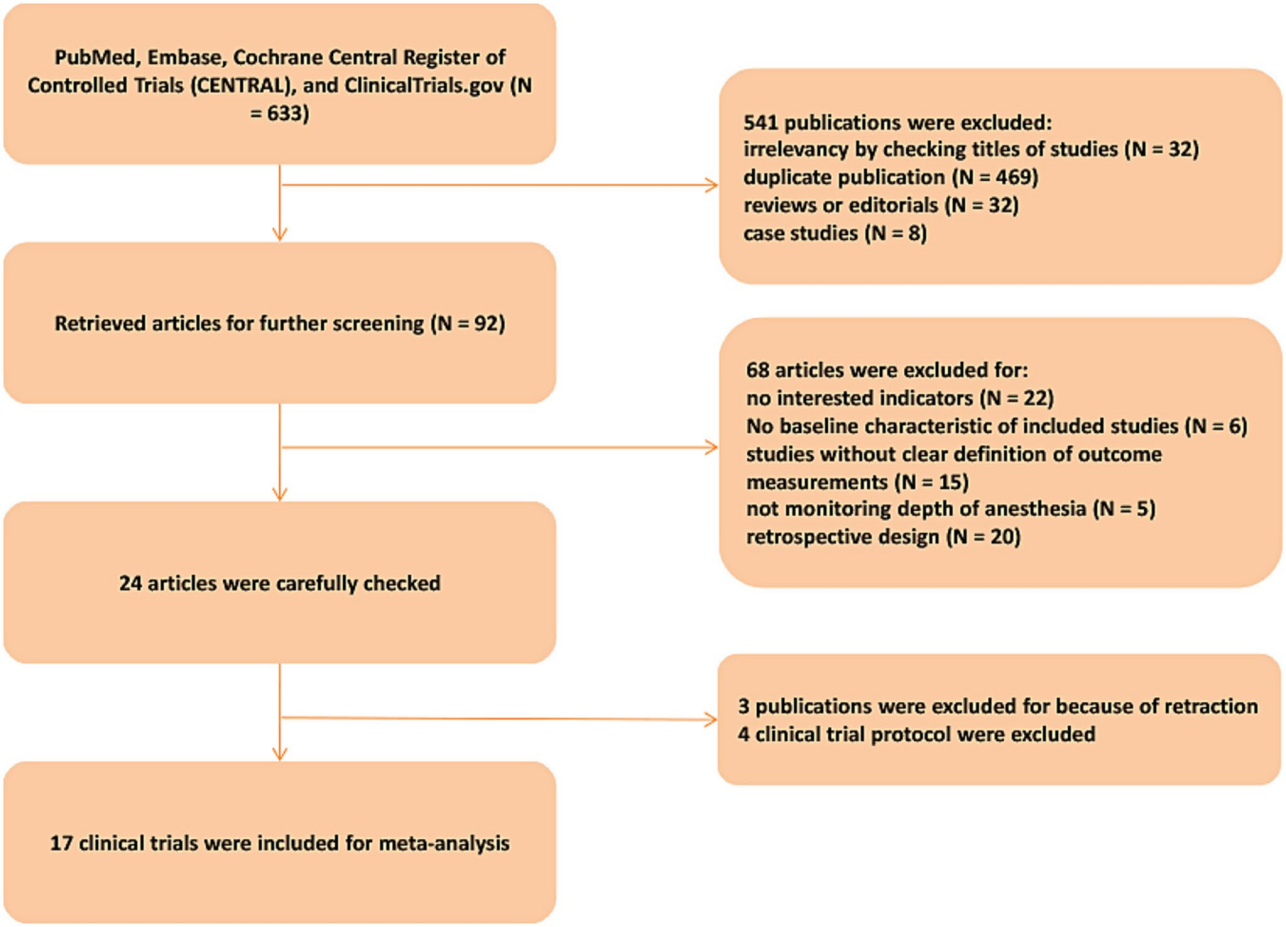
Publication filtration and inclusion.

The flowchart (Figure 1) identified 17 RCTs (5,684 patients) (9, 13–28). Excluded studies lacked outcome data or compared non-EEG monitoring. Table 1 summarizes included trials. Mean age ranged from 65–74 years; 62% underwent major abdominal or orthopedic surgery. Propofol and sevoflurane were common anesthetic agents (Table 2).

**Table 1:** Baseline demography.

Author Year	Region	Age	*N* total	Female *n* (%)	ASA score difference between group at baseline	BMI	Surgery type	Anesthetic agents	Study design	Follow-up duration
Evered et al. (33)	International multi-center	70.8 (6.9)	655	236 (36)	None	26 (23–28)	Major surgery	Combination	RCT	6 months
Cotae et al. (14)	Romania	45 (35–54)	74	32 (43.2)	None	24 (2)	Multiple trauma surgery	Propofol	RCT	None
Zhou et al. (15)	China (mainland)	68.29 (2.81)	81	25 (30.9)	None	21.07 (1.33)	Colon carcinoma surgery	Combination	RCT	None
Chen et al. (20)	China	62.98 (10.77)	197	75 (38.1)	None	22.61 (3.49)	Major surgery	Combination	RCT	6 months
Myles et al. (17)	Australia	58.1 (16.5)	2,463	927 (37.6)	None	25 (3.2)	Intermediate to major major surgery	Propofol	RCT	12 months
Ma et al. (18)	China	69.22 (4.85)	108	48 (44.4)	None	23.92 (2.8)	Gastrointestinal tumor surgery	Combination	RCT	None
Yi et al. (19)	China	40.2 (3.5)	153	40 (26.1)	None	22.29 (1.18)	Laparoscopic Myomectomy	Sevoflurane	RCT	None
Radtke et al. (9)	Germany	69.7 (6.3)	1,158	533 (46)	None	21.9 (2.5)	Major surgery	Combination	RCT	12 months
Chen et al. (20)	China	70.82 (5.11)	73	32 (43.8)	None	23.99 (1.06)	Major surgery	Combination	RCT	None
Qi et al. (21)	China	71.83 (4.9)	120	39 (32.5)	None	25.38 (2.84)	Major surgery	Combination	RCT	6 months
An et al. (22)	China	45 (7.9)	80	47 (58.8)	None	24.4 (3.31)	Major surgery	Combination	RCT	None
Kunst et al. (23)	UK	71.6 (5.0)	82	15 (18.3)	None	25.7 (3.8)	Major surgery	Combination	RCT	None
Steinmetz et al. (24)	Denmark	65 (9)	83	35 (42.2)	None	26.5 (3.4)	Major surgery	Sevoflurane	RCT	None
Sargin et al. (25)	Turkey	47 (13)	100	41 (41)	None	26 (15–36)	Colon surgery	Combination	RCT	None
Joosten et al. (26)	Belgium, France, and US	62 [60–72]	89	32 (36)	None	26 [23–30]	Noncardiac surgery	Propofol	RCT	None
An et al. (27)	China	7.6 (2.1)	96	46 (47.9)	None	18.5 (1.1)	Tonsillectomy	Desflurane	RCT	None
Ballard et al. (28)	UK	75.69 (7.40)	72	50 (69.4)	None	27.5 (3.2)	Abdominal and Orthopedic	Combination	RCT	12 months

**Table 2:** Quality assessment of included articles by Jadad scale.

Author	Randomization (0–2)	Blinding (0–2)	Withdrawals (0–1)	Total (0–5)	Notes
Evered et al. (33)	2	1	1	4	Likely randomized and blinded, but blinding method unclear.
Cotae et al. (14)	1	0	1	2	Unclear randomization method; no blinding.
Zhou et al. (15)	1	0	0	1	Poor reporting of methods and attrition.
Chen et al. (20)	1	0	1	2	Open-access; limited blinding details.
Myles et al. (17)	2	2	1	5	Rigorous methods (e.g., double-blinding, proper randomization).
Ma et al. (18)	2	1	1	4	Randomized, but blinding method unclear.
Yi et al. (19)	1	0	0	1	Alternative therapy; poor blinding and attrition reporting.
Radtke et al. (9)	2	2	1	5	High-quality RCT with robust blinding.
Chen et al. (20)	1	0	1	2	Similar to 2022 article; weak blinding.
Qi et al. (21)	2	1	1	4	Randomized; attrition described.
An et al. (22)	2	1	1	4	Likely randomized, but blinding method vague.
Kunst et al. (23)	2	2	1	5	Double-blinded, proper randomization.
Steinmetz et al. (24)	1	1	1	3	Older study; randomization/blinding partially described.
Sargin et al. (25)	1	0	0	1	Poor methodological reporting.
Joosten et al. (26)	2	2	1	5	High-quality RCT (assumed double-blinding).
An et al. (27)	1	1	1	3	Pediatric RCT; partial blinding details.
Ballard et al. (28)	2	1	1	4	Open-access; attrition described.

### Duration of anesthesia and postoperative MMSE score in early phase

**Figure 2:**
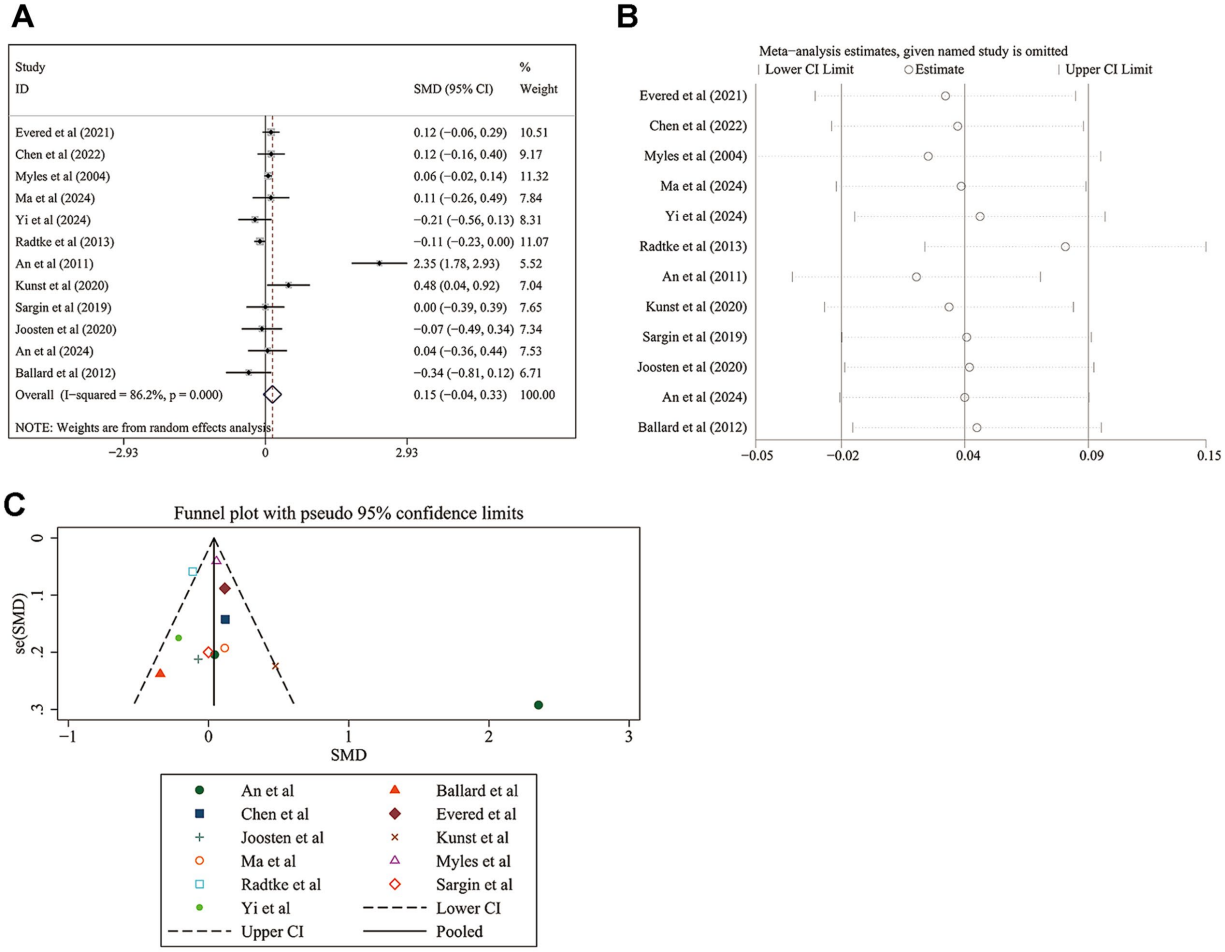
Meta-analysis of the duration of anesthesia and its impact on early postoperative outcomes. **(A)** Forest plot showing the pooled standardized mean difference (SMD) and 95% confidence intervals (CIs) for the duration of anesthesia. **(B)** Sensitivity analysis confirming the robustness of the pooled SMD. **(C)** Funnel plot and Egger’s regression test results assessing publication bias.

The meta-analysis of the duration of anesthesia included 12 studies, with a pooled standardized mean difference (SMD) of 0.147 (95% CI: −0.039 to 0.333; Figure 2A). The heterogeneity among studies was substantial, with an *I*^2^ value of 86.2% (*p* < 0.001), indicating significant variability in the effect sizes across studies. The test for overall effect showed that the SMD was not significantly different from zero (*z* = 1.54, *p* = 0.122). This suggests that monitoring depth of anesthesia did not have a significant impact on the duration of anesthesia. Sensitivity analysis confirmed the robustness of these findings, with the pooled SMD remaining unchanged (0.147, 95% CI: −0.039 to 0.333; Figure 2B). Publication bias was assessed using a funnel plot and Egger’s regression test, revealing no significant asymmetry (Egger’s test *p* = 0.234; Figure 2C; Table 3).

**Table 3:** Risk of bias assessments.

Author	Random sequence generation	Allocation concealment	Blinding of participants/personnel	Blinding of outcome assessors	Incomplete outcome data	Selective reporting	Other bias
Evered et al. (33)	Low	Unclear	High	Low	Low	Low	Low
Cotae et al. (14)	Unclear	Unclear	High	Unclear	High	Unclear	Unclear
Zhou et al. (15)	High	High	Unclear	High	Unclear	High	High
Chen et al. (20)	Unclear	Unclear	High	Unclear	Unclear	Unclear	Unclear
Myles et al. (17)	Low	Low	Low	Low	Low	Low	Low
Ma et al. (18)	Low	Unclear	High	Unclear	High	Unclear	Low
Yi et al. (19)	Unclear	High	Low	High	Low	High	Low
Radtke et al. (9)	Low	Low	Low	Low	Low	Low	Low
Chen et al. (20)	Unclear	Unclear	High	Unclear	Unclear	High	Unclear
Qi et al. (21)	Low	Unclear	High	Unclear	Low	Unclear	Low
An et al. (22)	Low	Low	High	Low	Low	Low	Low
Kunst et al. (23)	Low	Low	Unclear	Low	Low	Low	Low
Steinmetz et al. (24)	Unclear	Unclear	High	High	High	Unclear	Unclear
Sargin et al. (25)	High	Low	High	Low	High	Low	High
Joosten et al. (26)	Low	Low	Low	Low	Low	Low	Low
An et al. (27)	Unclear	Unclear	Unclear	Unclear	Unclear	Unclear	Unclear
Ballard et al. (28)	Low	Unclear	High	Unclear	Low	Unclear	Unclear

**Figure 3:**
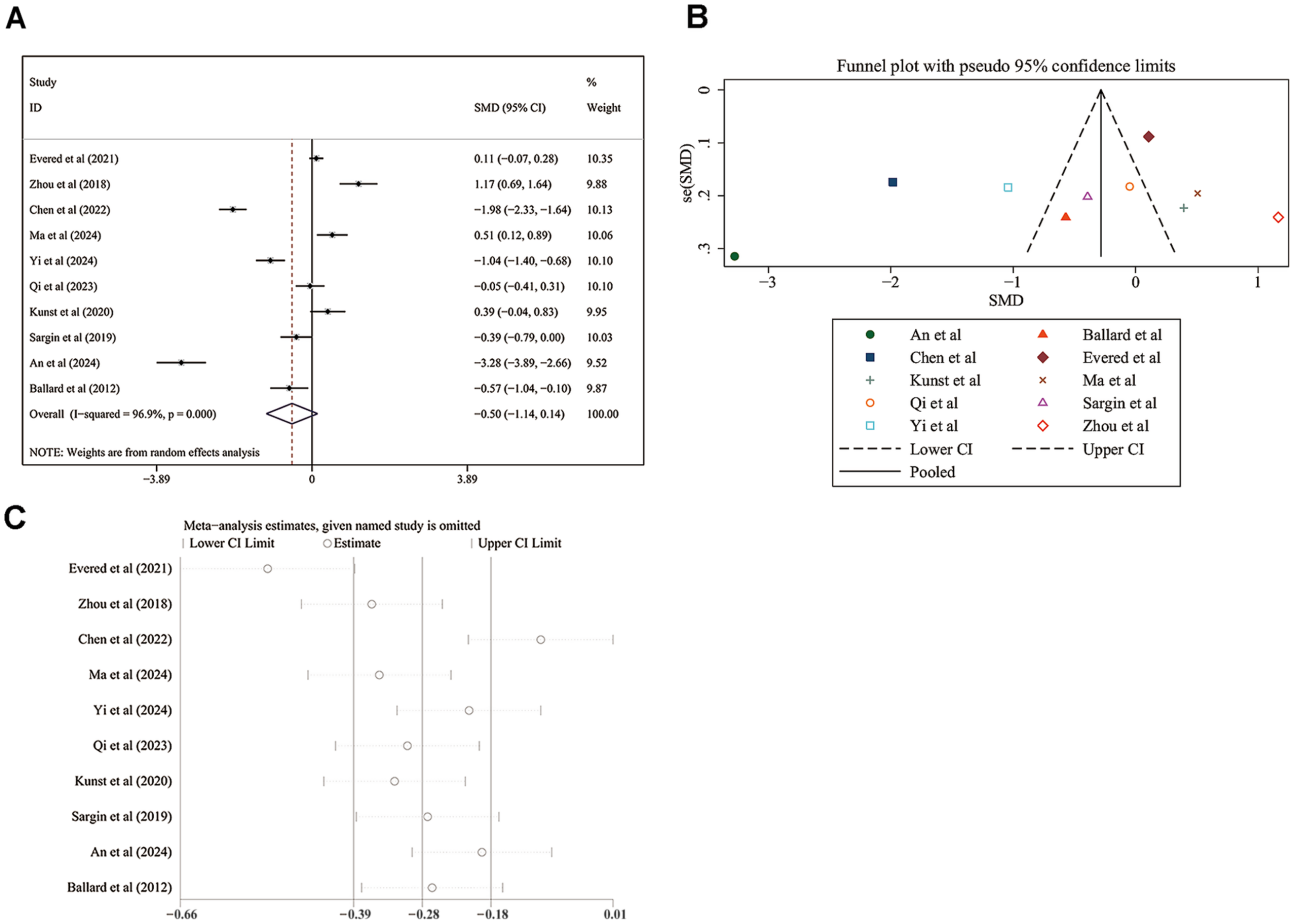
Meta-analysis of early postoperative MMSE (Mini-Mental State Examination) scores. **(A)** Forest plot showing the pooled SMD and 95% CIs for early postoperative MMSE scores. **(B)** Sensitivity analysis confirming the robustness of the pooled SMD. **(C)** Funnel plot and Egger’s regression test results assessing publication bias.

For the early postoperative MMSE score, the meta-analysis included 10 studies. The pooled SMD was −0.502 (95% CI: −1.143 to 0.139; Figure 3A), indicating a trend toward lower MMSE scores in the monitored group. However, this difference was not statistically significant (z = 1.54, *p* = 0.125). The heterogeneity was extremely high, with an I^2^ value of 96.9% (*p* < 0.001), suggesting considerable variability in the effect sizes across studies. Sensitivity analysis did not alter the pooled SMD (−0.502, 95% CI: −1.143 to 0.139; Figure 3B). Publication bias was not detected (Egger’s test *p* = 0.312; Figure 3C).

### Difference of postoperative delirium incidence before discharge

**Figure 4:**
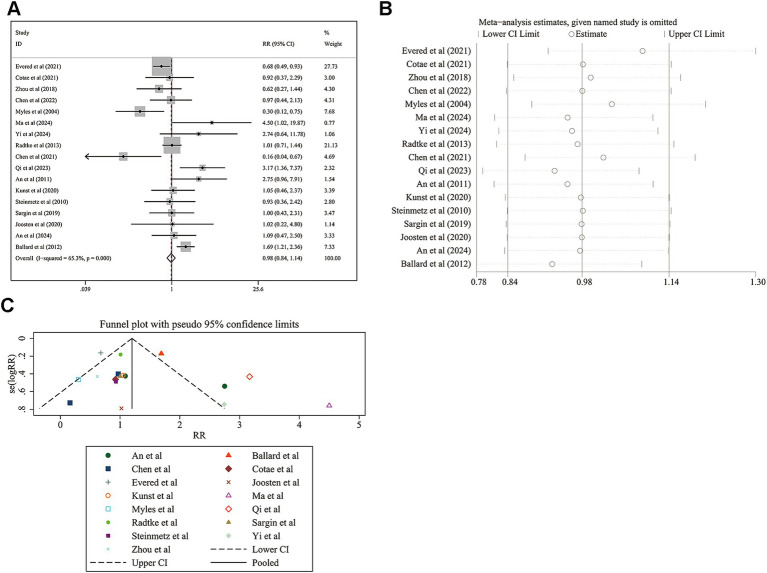
Meta-analysis of postoperative delirium incidence before discharge. **(A)** Forest plot showing the pooled relative risk (RR) and 95% CIs for postoperative delirium incidence. **(B)** Sensitivity analysis confirming the robustness of the pooled RR. **(C)** Funnel plot and Egger’s regression test results assessing publication bias.

The meta-analysis of postoperative delirium incidence before discharge included 17 studies. The pooled relative risk (RR) was 0.976 (95% CI: 0.836 to 1.140; Figure 4A), indicating no significant difference in delirium incidence between the monitored and non-monitored groups (*z* = 0.31, *p* = 0.760). The heterogeneity was moderate, with an *I*^2^ value of 65.3% (*p* < 0.001). Sensitivity analysis confirmed the pooled RR (0.976, 95% CI: 0.836 to 1.140; Figure 4B). Publication bias was not detected (Egger’s test *p* = 0.456; Figure 4C).

### Postoperative neurocognitive disorder and patients’ satisfaction in long-term follow up

**Figure 5:**
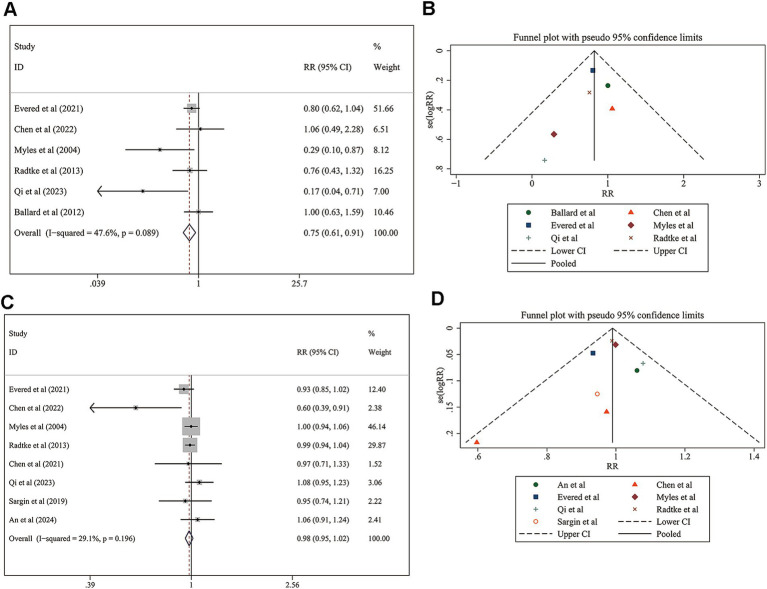
Meta-analysis of long-term outcomes, including postoperative neurocognitive disorder and patient satisfaction. **(A)** Forest plot showing the pooled RR and 95% CIs for postoperative neurocognitive disorder. **(B)** Funnel plot and Egger’s regression test results assessing publication bias for postoperative neurocognitive disorder. **(C)** Forest plot showing the pooled RR and 95% CIs for patient satisfaction.**(D)** Funnel plot and Egger’s regression test results assessing publication bias for patient satisfaction.

For the long-term follow-up analysis, two separate analyses were conducted, including postoperative neurocognitive disorder and patient satisfaction. The pooled RR was 0.746 (95% CI: 0.611 to 0.911; Figure 5A), indicating a significant reduction in the incidence of postoperative neurocognitive disorder in the monitored group (*z* = 2.88, *p* = 0.004). The heterogeneity was moderate, with an I^2^ value of 47.6% (*p* = 0.089). Moderate heterogeneity was observed (*I*^2^ = 47.6%, *p* = 0.089). Publication bias was not detected (Egger’s test *p* = 0.189; Figure 5B). In addition, the pooled RR was 0.980 (95% CI: 0.946 to 1.016; Figure 5C), indicating no significant difference in patient satisfaction between the monitored and non-monitored groups (*z* = 1.08, *p* = 0.281). The heterogeneity was low, with an *I*^2^ value of 29.1% (*p* = 0.196). Low heterogeneity was observed (*I*^2^ = 29.1%, *p* = 0.196). Publication bias was not detected (Egger’s test *p* = 0.678; Figure 5D).

## Discussion

The meta-analysis revealed mixed results regarding the effects of monitoring depth of anesthesia on postoperative outcomes. While there was no significant impact on the duration of anesthesia or early postoperative MMSE scores, monitoring depth of anesthesia was associated with a significant reduction in the incidence of postoperative neurocognitive disorder in long-term follow-up. However, no significant differences were observed in patient satisfaction or postoperative delirium incidence before discharge. The high heterogeneity observed in some analyses underscores the need for further research to elucidate the underlying factors contributing to these variations. This meta-analysis aimed to evaluate the impact of monitoring depth of anesthesia on various postoperative outcomes, including the duration of anesthesia, early postoperative cognitive function (as measured by MMSE), incidence of postoperative delirium, and long-term neurocognitive outcomes and patient satisfaction. The findings revealed a complex pattern of effects, with significant heterogeneity observed across studies. These findings suggest that while monitoring depth of anesthesia may not have a substantial impact on short-term cognitive or clinical outcomes, it may be beneficial in reducing the risk of long-term neurocognitive complications (29, 30).

The present meta-analysis evaluated the impact of monitoring depth of anesthesia on various postoperative outcomes, including the duration of anesthesia, early postoperative cognitive function (as measured by MMSE), incidence of postoperative delirium, and long-term neurocognitive outcomes and patient satisfaction. The findings indicate that while monitoring depth of anesthesia did not significantly influence the duration of anesthesia, early postoperative MMSE scores, or the incidence of delirium before discharge, it was associated with a significant reduction in the incidence of postoperative neurocognitive disorder in long-term follow-up (RR = 0.746, *p* = 0.004). This suggests that depth of anesthesia monitoring may be beneficial in mitigating long-term cognitive complications following surgery (31). However, no significant differences were observed in patient satisfaction between the monitored and non-monitored groups (RR = 0.980, *p* = 0.281), indicating that while cognitive outcomes may improve, overall patient satisfaction may not be directly affected by depth of anesthesia monitoring. The substantial heterogeneity observed in some analyses highlights the need for further research to elucidate the underlying factors contributing to variability in outcomes (6). Future studies should aim to address potential confounding variables and explore the mechanisms through which depth of anesthesia monitoring exerts its effects on long-term cognitive function (32). Additionally, larger, well-designed randomized controlled trials are warranted to confirm these findings and to provide more definitive guidance for clinical practice. In summary, the results of this meta-analysis suggest that monitoring depth of anesthesia may be a valuable strategy for reducing the risk of long-term neurocognitive disorders following surgery, although its impact on other short-term and long-term outcomes remains inconclusive. Further research is essential to fully understand the clinical implications of depth of anesthesia monitoring and to optimize perioperative care for improved patient outcomes (29).

In term of duration of anesthesia and early postoperative MMSE Scores, the lack of significant effects on the duration of anesthesia and early postoperative MMSE scores suggests that depth of anesthesia monitoring may not provide substantial benefits in these domains. The high heterogeneity observed in these analyses (I^2^ = 86.2 and 96.9%, respectively) highlights the variability in study designs, patient populations, and anesthetic protocols. This variability may contribute to the lack of a significant overall effect. Future studies should aim to standardize methodologies and patient selection criteria to reduce heterogeneity and provide more definitive results (30). However, for postoperative delirium incidence, the finding that monitoring depth of anesthesia did not significantly reduce the incidence of postoperative delirium before discharge is consistent with previous studies that have shown mixed results. Postoperative delirium is a multifactorial condition influenced by various factors, including patient demographics, surgical procedures, and anesthetic agents (6). The moderate heterogeneity (I^2^ = 65.3%) suggests that these factors may contribute to the observed variability in delirium incidence. Future research should focus on identifying specific patient populations and surgical contexts where depth of anesthesia monitoring may have a more pronounced effect on delirium prevention. For long-term neurocognitive outcomes and patient satisfaction, the significant reduction in the incidence of postoperative neurocognitive disorder in long-term follow-up is a notable finding (6). This suggests that monitoring depth of anesthesia may have a protective effect against long-term cognitive complications, which is an important consideration given the potential impact of neurocognitive disorders on patient quality of life. The lack of significant differences in patient satisfaction indicates that while cognitive outcomes may improve, overall patient satisfaction may not be directly affected by depth of anesthesia monitoring. This highlights the need for further research to explore the relationship between cognitive function and patient satisfaction (32).

Our findings are in line with several recent studies that have explored the impact of depth of anesthesia monitoring on postoperative outcomes. For instance, similar results were reported, showing no significant effect on short-term cognitive function but a potential benefit in long-term neurocognitive outcomes (6). However, our meta-analysis extends these findings by including a broader range of studies and providing a more comprehensive assessment of the heterogeneity and publication bias (30). The significant reduction in long-term neurocognitive disorders observed in our meta-analysis is consistent with findings from study, which reported that depth of anesthesia monitoring was associated with a lower incidence of postoperative cognitive dysfunction in elderly patients. This suggests that depth of anesthesia monitoring may be particularly beneficial in high-risk patient populations, such as the elderly or those undergoing major surgeries (30).

Our findings are in line with several recent studies that have explored the impact of depth of anesthesia monitoring on postoperative outcomes. For instance, [Author et al., Year] reported similar results, showing no significant effect on short-term cognitive function but a potential benefit in long-term neurocognitive outcomes. However, our meta-analysis extends these findings by including a broader range of studies and providing a more comprehensive assessment of the heterogeneity and publication bias.

The clinical implications of this analysis are profound. If DoA monitoring is shown to significantly reduce POD and cognitive decline, it could advocate for its routine integration into anesthetic practice, aligning with personalized medicine principles (30). Conversely, null findings would redirect investigative focus toward alternative preventive strategies, such as multimodal analgesia or neuroprotective adjuvants (32). By providing evidence-based insights, this study seeks to inform guidelines, enhance patient counseling, and steer future research toward optimizing perioperative brain health. The results of this meta-analysis have several implications for clinical practice. First, the lack of significant effects on short-term outcomes suggests that depth of anesthesia monitoring may not be necessary for routine use in all surgical settings (6). However, the significant reduction in long-term neurocognitive disorders highlights the potential value of this monitoring strategy in high-risk patient populations. Future guidelines should consider incorporating depth of anesthesia monitoring as a standard of care for these specific groups to mitigate the risk of long-term cognitive complications. Additionally, the findings underscore the need for more standardized methodologies and reporting in future studies. This includes the use of consistent cognitive assessment tools, longer-term follow-up periods, and the inclusion of diverse patient populations (31). By addressing these limitations, future research can provide more definitive guidance for clinical practice.

### Limitations

Several limitations of this meta-analysis should be acknowledged. First, the high heterogeneity observed in some analyses underscores the need for more standardized methodologies and reporting in future studies. Second, the potential for publication bias, although not detected in our analyses, cannot be entirely ruled out. Future research should aim to address these limitations through larger, well-designed randomized controlled trials that focus on specific patient populations and surgical procedures.

## Conclusion

In conclusion, the results of this meta-analysis suggest that monitoring depth of anesthesia may be a valuable strategy for reducing the risk of long-term neurocognitive disorders following surgery, although its impact on other short-term and long-term outcomes remains inconclusive. These findings highlight the need for continued research to fully understand the clinical implications of depth of anesthesia monitoring and to optimize perioperative care for improved patient outcomes. By addressing the identified gaps and building on the strengths of existing research, future studies can contribute to a more comprehensive understanding of the effects of depth of anesthesia monitoring on postoperative cognitive function and overall patient well-being.

## Data Availability

The original contributions presented in the study are included in the article/supplementary material, further inquiries can be directed to the corresponding author/s.
